# Analysis of Network Structure and Doctor Behaviors in E-Health Communities from a Social-Capital Perspective

**DOI:** 10.3390/ijerph17041136

**Published:** 2020-02-11

**Authors:** Zhigang Li, Xu Xu

**Affiliations:** 1 School of Management Science, Chengdu University of Technology, No. 1, Dongsan Road, Erxian Bridge, Chenghua District, Chengdu 610059, China; cdlglzg@163.com; 2 Chengdu University of Technology, No.1, Dongsan Road, Erxian Bridge, Chenghua District, Chengdu 610059, China

**Keywords:** social network analysis, information dissemination behavior, social capital, e-health community, interaction frequency

## Abstract

In tandem with internet development and widespread social media use, e-health communities have begun to emerge in recent years. These communities allow doctors to access forums anywhere, anytime, seek or exchange medical information online, find literature, and so on. This is convenient and can solve some problems for doctors while also promoting doctor communication. This study collected and collated 102 doctors in the “Lilac Forum” and used social network tools to quantify the overall network density, centrality, core–periphery structure, and structural hole indicators of doctors’ information exchange from a social-capital perspective. The results showed that the frequency of interaction between doctors differed because of differences in the identities and participation of doctors in the e-health community. The density of the doctors’ information dissemination network (0.228) and network cohesion (0.610) were relatively high. Thus, the doctors were more closely connected, and information was easily spread. At the same time, doctors with higher professional titles had obvious location characteristics, familiarity and trust, and high levels of reciprocity. They could obtain redundant information in the network and were more likely to influence the behavior of other doctors. This study’s findings provide support for improving information exchange among doctors in e-health communities and improving the service levels of the platforms.

## 1. Introduction

The issue of resource sharing has attracted a great deal of attention. Given the imbalances in the distribution of medical resources in developing countries, medical information sharing has received considerable attention. China is a vast country with a large population, and the development of medical standards in urban and rural areas is uneven [1]. This is a longstanding problem in China. With rapid internet development and widespread social media use, online medical resources have gradually emerged [2], offering a potential solution to the aforementioned problems. In the traditional medical mode, information provision takes place via consultation between doctors and patients. Doctors mainly diagnose and treat through discussion, notification, and interpretation [3,4,5]. Today, doctors’ demands for medical information have increased, and patients are likewise eager to get more information and opportunities [6]. With the support of policies, many e-health communities have emerged in recent years. Such communities provide platforms for communication in the areas of medicine, pharmacy, life sciences, and other related fields. These are professional social networks for doctors, medical institutions, and medical practitioners [7]. Forums such as Lilac (www.dxy.cn/bbs) and Love Love Doctor (bbs.iiyi.com/) provide medical information and services to many doctors. However, there are still relatively few active users in medical forums [8], which means the acceptance level of e-health communities is still lacking. Therefore, it is important to explore the information exchange behaviors of doctors in such communities to promote the service level of e-health communities.

The e-health community is an electronic information platform where doctors can exchange academic, basic medical, and other information. In e-health communities, doctors can access forums anywhere, anytime, seek or exchange medical information, find literature, and so on. At the same time, doctors can also post comments and questions on the forum and communicate with others. This is convenient and can solve some problems for doctors. If a doctor’s area lacks the advanced medical resources and medical resources, and a patient requires advanced treatment, the doctor can access relevant advanced medical information in a forum. Then, the patient can be better prepared for the advanced treatment before the referral, which can increase cure rates [9]. E-health communities also can break the limitations of time and space and help doctors obtain professional knowledge from other doctors at different levels, which can reduce imbalances in medical resources [10]. Although such advantages indicate that e-health communities have good prospects, some shortcomings still exist. First, users of the Lilac forum are independent and scattered [11], making it difficult to establish good relationships between doctors, which can lead to redundant or complex information. Second, users might think that using the e-health community entails certain risks [12] and may have reservations about the relevance of the medical information. Finally, users sharing information in the community cannot disseminate this information in a timely and rapid manner, which may reduce information quality [13]. Such shortcomings can have negative effects on the development of e-health communities. Therefore, this study aimed to use social network analysis to analyze doctors’ behaviors in e-health communities to provide support for e-health services.

Many studies [14,15,16,17,18] have investigated the exchange of health information in online groups, finding abundant information environments in such communities. At the same time, the behavior of information in online communities can change based on the composition of community members and the nature of the benefits [19]. Social networks are as important as other mechanisms for improving healthcare opportunities [20]; therefore, it is feasible to use social networks to analyze online networks. Social network analysis includes the following metrics: density, centralization, geodesic distance, centrality, core–periphery, and structural holes. Jingyuan Shi and Charles T Salmon analyzed the centrality and shortcut distance of social media information interaction networks [21], while Jing yuan Shi, Xiaohui Wang, Tai-Quan Peng, et al. increased the density index [22]. Based on such work, the present study also increased the core–periphery structure and structural hole indicators. Moreover, it further analyzed the social network, which can effectively supplement the theoretical part of the network structure index and thus has a certain value. Social capital is generated and measured in the network. However, there is a gap in the literature regarding the integration of social capital and network structure. Therefore, this study’s analysis of the e-health community in combination with the above methods is also innovative. The purpose of this study was to describe the behaviors of doctors in electronic medical communities and explore their status, influence and level of social capital in the networks. The results can provide a channel for medical information resource sharing and support the development of online medical care. 

## 2. Materials and Methods

### 2.1. Description of the Sample

This is mainly a descriptive study. The sample came from the Doctor Information Sharing Forum—the Lilac Forum (a subsidiary of Lilac Garden). We started collecting relevant sample data from April 2019, which lasted for two months. It mainly collects doctors who are active in the Lilac Forum neurology column and rank higher on the list of activities. A total of 102 doctors were collected and were represented by 102 assigned numbers. These users in neurology are related nodes. They came from different provinces and different grades of hospital, and they had different titles. Thirty-four had primary titles (healer, physician, resident), 33 had intermediate titles (attending physician), 27 had deputy senior titles (deputy chief physician), and 8 had senior professional titles (chief physician). Table 1 shows the specifics. The inclusion in this study of doctors with different professional titles ensured that the study was representative of the information exchanged in the forum.

### 2.2. Instruments Used to Collect Data and Variables

Data collection was conducted through the Lilac Forum, which includes a clinical medicine area, a study and examination area, an information exchange area, a basic public health area and so on. There are more than 100 columns in all areas for doctors to discuss various topics. In these columns, doctors can obtain the experimental technical discussion and professional knowledge exchange services provided by the Lilac Forum. The main survey content concerned whether doctors communicated and shared information in neurology columns. We obtain the information exchange situation between neurologists in the Lilac Forum, and construct a “0–1” matrix of doctor information exchange adjacency relationships; a 1 means “the two have generated information interaction behavior,” and a 0 means “the two do not generate information interaction behavior.” Individuals were coded using numbers to ensure privacy. The cohesion variable was described using the following metrics: density, centralization, geodesic distance, centrality, core–periphery, and structural holes (Table 2). Both Ucinet and Gephi are tools for social network analysis. Ucinet is more suitable for data operations than Gephi. It supports a large number of algorithms and can make accurate calculations and analysis of matrices. Gephi can draw more clearly diagram than Ucinet [23]. Therefore, Ucinet is used to find the relevant metrics for analysis, and Gephi is used to drawn the network diagram.

### 2.3. Definition and Measurement of Social-Capital Theory

Generated social capital can improve the quality and quantity of information exchange in a forum [19], increase learning opportunities, and promote knowledge exchange [30]. Social capital that individuals do not directly possess or use is embedded in social networks; it can be approached and used by being a member of the network or establishing a network connection. Therefore, a medical network can be effectively measured by social-capital theory. The ability of individuals to acquire social capital through the internet will be enhanced, and social capital as a resource will promote people’s further development, thus forming a virtuous circle. Social-capital theory, as proposed by Granovetter, is used to measure a constructed network and is mainly based on four indicators: interaction frequency, emotional density, familiarity between subjects or mutual trust, and reciprocal exchange [31]. Therefore, we will further explain the doctor information interaction network according to social-capital theory.

Interaction frequency. This refers to the degree of participation of each subject in the medical network [32]. As long as they agree with the doctor, they will collect these posts and hit the like button. If there are diverse opinions on the posts, doctors can comment and even vote on different opinions to express their opinions. The number of visits, votes, and collections of medical information published by the 102 doctors in the sample was calculated by subtracting the value at the beginning from the value at the end of data collection. These three sets of data were measured using the following metrics: average, median, moderate, standard, kurtosis, skewness, maximum, and minimum. Among them, standard, kurtosis and skewness are used to measure the dispersion of sample values.

Emotional density. The higher the emotional density, the closer the connections between individuals in the network. This can effectively reveal information communication relationships in the e-health community [33]. It is measured by density, centralization, and geodesic distance.

Familiarity between subjects or mutual trust. The higher the coefficient, the easier it is for the individual to influence the behaviors of other doctors [34]. It is measured by core–periphery mode and centrality.

Reciprocal exchange. This refers to the information redundancy that occurs when an individual transmits information in the network that can generate certain benefits. The individual obtains information that other doctors cannot obtain, and the degree of reciprocity is high. This can effectively control the dissemination of information and bring benefits such as job promotions, wage increases, and increased prestige [28]. It is measured by structural holes.

## 3. Results

### 3.1. Network Analysis

**Figure 1:**
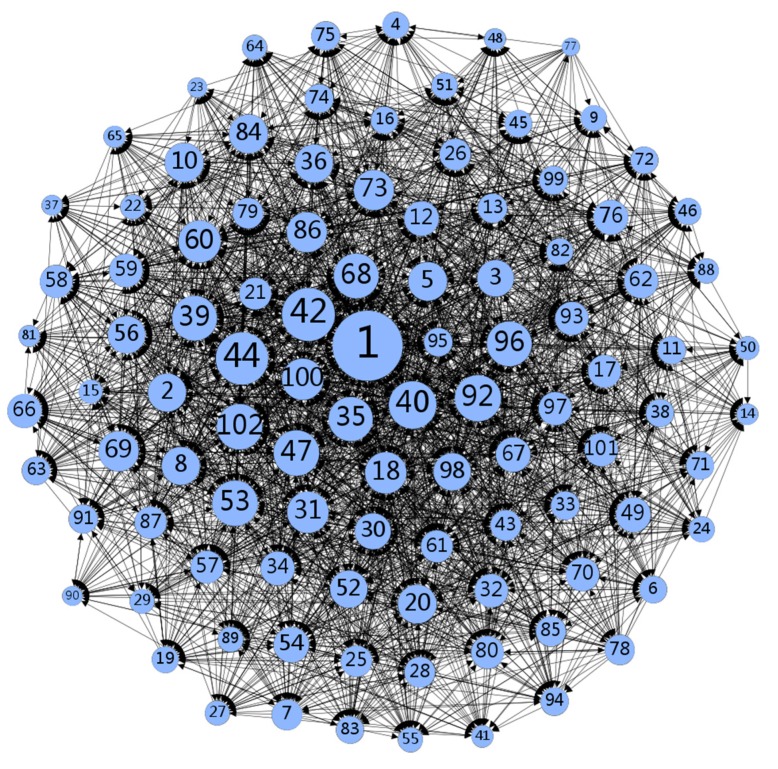
Doctor information interaction network diagram.

Data were collected using Excel and were examined according to demographics and by a sociogram. In Figure 1, created using Gephi, the nodes mainly rely on the degree of centrality in the data. Round nodes indicate doctors, while larger circular shapes represent individuals with a high degree of centrality. Nodes represent the actors in the communication network, lines represent the relational ties in information seeking, and arrows indicate the direction of information seeking. Figure 1 shows that there are no isolated nodes in the network, indicating that doctors are active and relatively close in the e-health community. At the same time, some doctors are at the center and are closely connected with other nodes.

### 3.2. Interaction Frequency

Table 3 shows that the average number of views is 80,362, while the number of collections only accounts for 8.99% of the number of views and the three groups of data all have large standard deviations. This indicates that the frequency of interactions between doctors has a significant difference. In addition, the skewness is a small positive number, meaning that the value distribution has a short right tail. Therefore, a few doctors have very high numerical values in the three sets of data and have a higher frequency of interaction and sense of identity. The differences between the maximum and minimum of the three sets of data was 541,639, 192,431, and 104,230, respectively. The extreme difference is high, indicating that the cognitive status of each doctor in the medical community is different, and the frequency of interaction is different. In Table 4, these doctors (individuals 40,1,62) have the highest number of views, votes and favorites. According to subsequent analysis, we found that they also occupy the core position. Not only did their social capital increase during the interaction, they also affected other doctors.

### 3.3. Emotional Density

#### 3.3.1. Density and Centralization

The density of the network is 0.228. In general, the overall network is relatively close, information interaction between doctors is relatively close, and emotional density is high. Table 5 shows that degree centralization is relatively high, there are some key node doctors, and information dissemination is transmitted from key node doctors to other doctors. However, betweenness centralization is low, indicating that doctors play a certain controlling role in the process of information dissemination; however, the effect is small.

#### 3.3.2. Geodesic Distance

As seen in the Figure 1, all nodes in the network are reachable, with an average geodesic distance of 1.800, which means that any doctor in the e-health community can communicate with other doctors on average after nearly two doctors. Meanwhile, the cohesiveness of the network is 0.610, and there are no isolated nodes in the network diagram, indicating that the network has strong cohesion.

### 3.4. Familiarity between Subjects or Mutual Trust

#### 3.4.1. Centrality

In Table 6, some individuals (2, 3, 10, 39, 40, 42, 84, 86) have a larger out-degree and smaller in-degree of Freeman’s degree and of closeness. This means other doctors in the community are strongly affected by these doctors’ behaviors and are at the core of the network with a certain influence and a high level of familiarity and trust.

Some nodes (e.g., individuals 1, 8, 31, 35, 36, 44, 47, 53, 56, 60, 68, 69, 73, 92, 96, 100, 102) have a large Freeman’s betweenness. This shows that these doctors are directly linked with other doctors, and information exchange is frequent. In the information dissemination network, the node (i.e., the individual) is in the subcore position.

#### 3.4.2. Core–Periphery Model

**Figure 2:**
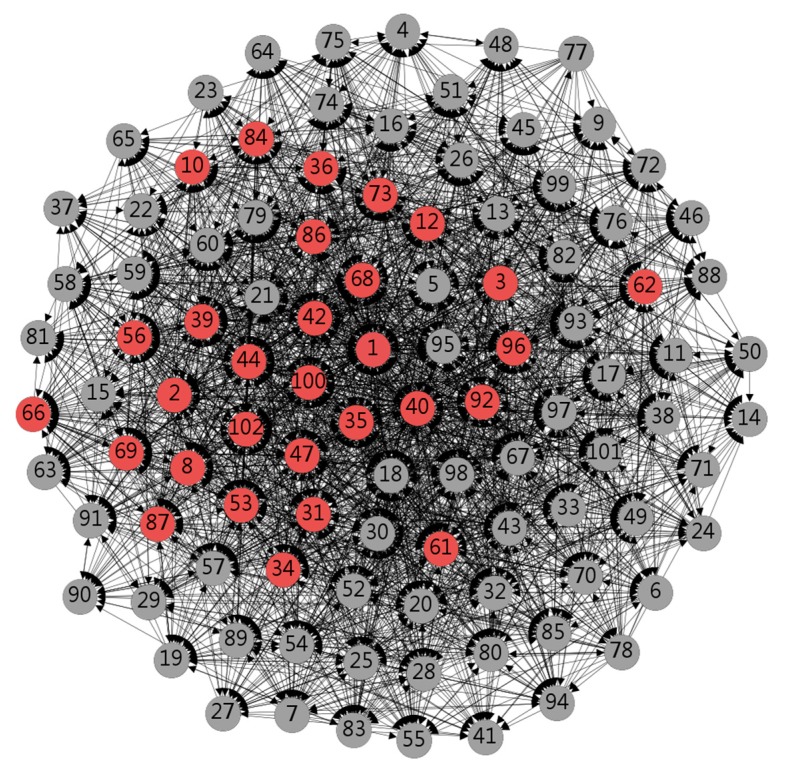
Doctor information interactive network core–periphery structure.

Figure 2 shows the core–edge properties of the data. Round boxes represent individual doctors, red represents individuals with core attributes, and gray represents individuals with edge attributes. Among them, some doctors (individuals 1, 2, 3, 8, 10, 12, 31, 34, 35, 36, 39, 40, 42, 44, 47, 53, 56, 61, 62, 66, 68, 69, 73, 84, 86, 87, 92, 96, 100, 102) are in the core area. According to the calculation results, the core area network density in the network is 0.455, and the periphery area network density is 0.181. The periphery area network density is lower than the core area network density and is also smaller than the overall network density. By comparison, the density of the core area network is greater than the overall network density, which is related to the core position of the behavioral leader.

### 3.5. Reciprocal Exchange

**Figure 3:**
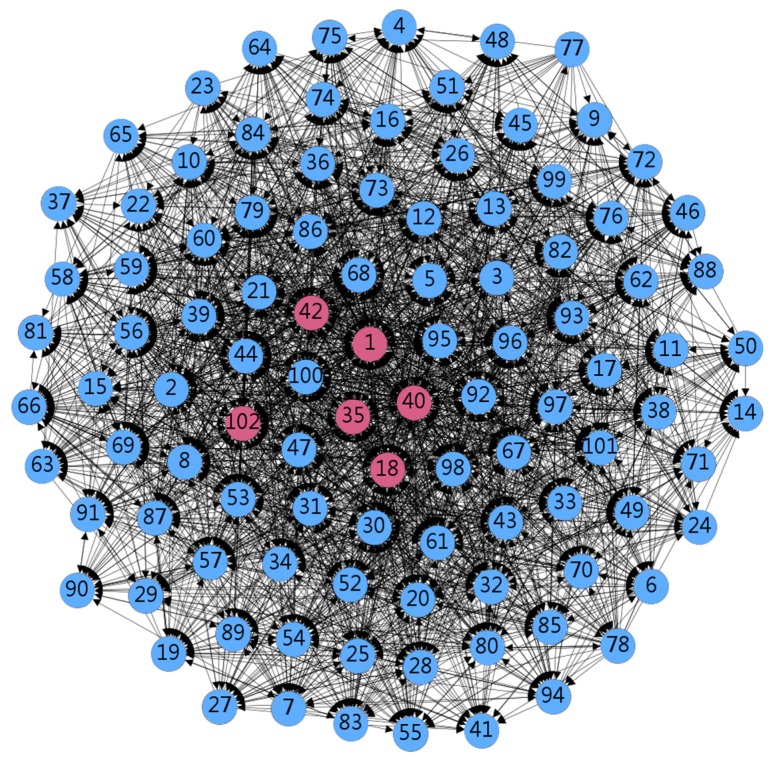
Doctor information interaction network structural hole analysis diagram.

Since the core and periphery area networks have significant differences, there are “structural hole” structures that can be connected to the two areas. Based on the above analysis, some doctors (individuals 1, 3, 18, 31, 35, 39, 40, 42, 44, 73, 92, 96, 100, 102) have a large intermediate center, indicating that these 14 nodes are likely to occupy the structural hole position. Additional indicators are needed for verification. By comparing effective size, efficiency, and constraint, it can be found that individuals 1, 18, 35, 40, 42, and 102 are located in the structural hole of the network (i.e., the middleman of the network), and the degree of reciprocity is high. Doctors in such nodes can connect to two areas, or they can bring location benefits through a reciprocal relationship with the structural hole location (Figure 3). Figure 3 shows the properties of the structural holes in the data. A red round box represents an individual with structural hole properties. The solid lines with arrows connecting individuals represent information interactions between them.

## 4. Discussion

### 4.1. Sense of Identity in the Network is Positively Related to Participation

It is generally believed that individuals will use the forum again or even more when they receive more replies, comments and likes in the e-health community. This kind of reply, comment and collection is defined as sense of identity in the text. the more you get from the above behaviors, and the more your sense of identity will increase accordingly. In the analysis of Table 4 and Figure 2, it was found that the three doctors with the highest number of views, votes and favorites all occupied the core position. So, sense of identity in the network is positively related to participation. A sense of identity in social networks can give individuals a strong sense of belonging and promote their appreciation of network relationships [35]. In this study, interaction between doctors was expressed by the frequency of interaction. The interactive frequency analysis indicated that differences in external factors, such as the platform time, caused the number of visits, votes, and collections to be different, leading to differences in doctors’ sense of identity in the community. Furthermore, a strong sense of social identity produces a network of mutual support [36], which gives participants access to information and emotional support [37]. Identity differences create imbalances in the development of e-health communities. Since some users with lower interaction frequency do not find value in their participation, they will gradually withdraw from the community. By contrast, doctors with a higher sense of identity will actively participate and play a mutually reinforcing role. This also validates previous study conclusions that the sense of identity in the community will increase individual participation [38]. From the perspective of social-capital theory, for doctors with lower interaction frequency, there should be a new section to increase the number of views and collections, thus increasing their sense of identity to maintain its existing users. There are significant differences in the frequency of interaction between doctors, which in turn gives rise to different senses of identity among doctors in the online community. Simultaneously, individuals in the community will have interdependent emotions and will have more social identity, especially emotional identity. Therefore, the new users of the e-health community should be encouraged to actively participate in the exchange of information and gain the approval of other doctors. Cooperation between doctors can also be enhanced and services such as information provision and emotional attribution of the individuals in the e-health community can be strengthened to increase information exchange between doctors.

### 4.2. Close Communication of Information in the E-Health Community

Along with increased internet penetration [39], people are increasingly obtaining medical knowledge from online communities and discussing health issues online to manage their own health [40,41]. Figure 1 shows that doctors interact with information in medical online communities. Communication in the community covers many areas, not just medicine [42,43,44,45,46]. In the process of information exchange, different patients and doctors might need different types and quantities of information [47]. Doctors in the community conduct information interactions related to all aspects of medical care on an as-needed basis. In the doctor information interaction network, the level of knowledge and behavior is high. The overall doctor information interaction behavior network is relatively tight. Its geodesic distance is small (1.800), the information has high accessibility, information interaction between doctors is relatively close. There is no isolated node, and the doctors were closely connected. It is different from previous research conclusions [6,48], which may be due to the different nature and content of the selected e-health community. According to the research data in this paper, information is easily spread in the e-health community. Some doctors have higher emotional density, which in turn can garner a higher reputation, more information, and other rewards. Communities with close information flows also provide a basis for better service to individuals.

### 4.3. Significant Positions of Doctors with Higher Professional Titles

(1) Doctors with higher professional titles might appear in the core position

Individuals in the core position are dominant in the exchange relationship [49] and are more important in the online community than in the periphery areas [50]. A network with a core–periphery structure consists of densely interconnected core nodes. Core nodes can be connected to periphery nodes, while periphery nodes cannot be densely interconnected [51]. This study found a core–periphery structure in the medical network, and an individual’s personal attributes are significant. In addition to individual 18, the other seven doctors in the core area had senior titles and were at the core position. Among those with deputy senior titles, 12 doctors were at the core. Therefore, doctors with higher professional titles have a greater chance of appearing in the core position, and their information interaction behavior has an important effect on other doctors in the community. This is consistent with recent study that doctors with different titles have different performances, and doctors with higher professional titles have higher visibility in online communities [52]. At the same time, the network density at the core of the network was relatively high (0.455), indicating that the overall network of information interaction behavior was greatly affected by doctors in the core area. Doctors with lower professional titles could be periphery because of other factors, such as their own capabilities. Doctors in the structural hole position have high levels of familiarity, trust, and reciprocity, which in turn lead to higher social capital, and doctors at the periphery are affected by them. Identifying these individuals can effectively control the dissemination of information. They can manage the e-health community in an orderly manner, promote information flow between doctors, improve cooperation, communicate advanced medical information, and conduct academic exchanges to resolve medical problems caused by geographical differences. Simultaneously, enhancing the service functions of the e-health community, such as information retrieval and information popularization, will help doctors at different levels to obtain higher social capital.

(2) Senior title doctors occupy the structural hole position

A network with structural holes can more easily acquire and disseminate new information [53]. Doctors in structural holes are more likely to receive redundant information in the network [54] with a higher level of reciprocity. In community leadership and prevention strategies, network analysis can effectively identify key players as well as others in the network [27]. Individuals 1, 18, 35, 40, 42, and 102 were in the structural hole position in the network. The existence of structural holes allows occupants the opportunity to provide access to “information benefits” and “controls”, which creates more competition than members in other locations in the network. Structural holes can effectively connect the entire network. These findings are consistent with previous study [55]. As shown in Figure 3, the six doctors occupying the structural hole (of which five have senior titles) have a high degree of reciprocity, and are behavior leaders in the community. That person can obtain more redundant information, avoid information occlusion, and effectively spread information. Identifying doctors occupying structural holes can promote the dissemination of information, and at the same time, the doctor’s social capital continues to increase during this information dissemination process. Promoting information dissemination can also promote the development of the entire medical network platform. 

### 4.4. Ethics and Data Privacy

With the development and utilization of medical data, many medical resource data sharing platforms have been established, which has improved the utilization efficiency of medical resources [56]. However, the existing sharing platforms and technologies have defects in privacy protection [57]. For example, behaviors such as doctors talking about patients and asking colleagues’ opinions, collecting data exchanged between doctors in Lilac e-health community all involve ethical issues of medical data privacy. However, because there is no relevant ethics review agency in China, and the current regulations do not require an ethics review of such research, the study has not been subjected to approval by an Institutional Review Board. Furthermore, although there is no sound data privacy law in China that clearly indicates which data of forum users is involved in infringement, in order to protect the privacy of participants, we have adopted two protection methods in accordance with basic privacy protection regulations: first, we adopt the method of anonymously collecting data, and only collect the public information of Lilac Forum, such as the doctor’s title information and the degree of activity in the forum. Secondly, when collecting data on doctors’ information exchange, we only collect whether there is communication between doctors and do not obtain the specific content of the communication. However, it is worth noting that with the continuous development and progress of China’s new data privacy law in the future, these laws and regulations will change the information exchange behavior of doctors in the e-health community, and may greatly change or affect the research conclusions of this article. In response to these medical ethics and private data protection issues, many scholars have proposed relevant countermeasures: strengthening the subject’s awareness of privacy and security, upgrading data privacy protection technology, improving social restriction systems [58], and informing policy makers to reform laws and policies [59].

## 5. Conclusions

As an interactive tool, a social network can communicate information to better serve the community and benefit health promoters [60]. Analyzing social networks that disseminate medical information can help to improve doctors’ information exchange between e-health communities. In this research, we constructed a social network based on a social-capital perspective to describe the social capital and information exchange behavior of doctors in the e-health community and explore its position in the network. We also studied the overall network structure of information exchange in the e-health community. The conclusions are summarized below.

(1) In the Lilac e-health community, the closeness and degree centralization of the doctor’s information interaction network is relatively high, the doctor’s information exchange is close, and the information is easily spread on the network.

(2) There is a core–periphery structure in the network. Doctors with a senior professional title or a deputy senior title occupy core position of the network. These doctors have higher social capital, and their behavior and development affect doctors in the periphery position.

(3) Senior title doctors occupy the structural hole position, and have high levels of familiarity, trust, and reciprocity, thereby having higher social capital. These doctors can receive two kinds of information in the network and have the function of regulating and controlling the network information.

Based on the results of the data analysis, we suggest that the Lilac Forum can introduce topical posts such as hot topics and classic medical case discussions to increase the frequency of doctor interaction in the forum and the participation of doctors at different levels. Additionally, we recommend that doctors in any position in the e-health community network can increase their social capital to a certain extent by increasing their participation in information exchange. There is still room for improvement in our study. For instance, only doctors in the neurology column were selected for data investigation and analysis in this article, but the medical forum has many columns, and doctors of different specialties may have different needs for information exchange. Therefore, in the future research, we can cooperate with the Doctor Lilac Forum platform to collect active users of all columns as samples, so as to better analyze and reflect the network structure, behavior mode and activity of doctor information exchange between different professional disciplines in the e-health community.

## Figures and Tables

**Table 1:** Physician title.

Job Title	Assigned Number
Primary title	2, 4, 5, 10, 15, 16, 17, 30, 33, 37, 38, 41, 43, 45, 50, 51, 52, 54, 55, 57, 63, 64, 65, 67, 81, 82, 83, 85, 87, 90, 91, 92, 93, 94
Intermediate title	3, 7, 9, 11, 12, 21, 22, 26, 27, 28, 29, 31, 36, 39, 44, 46, 48, 56, 68, 69, 71, 72, 74, 75, 78, 79, 80, 88, 89, 95, 97, 98, 101
Deputy senior title	6, 8, 13, 14, 19, 20, 23, 24, 25, 32, 35, 47, 49, 53, 58, 60, 61, 66, 70, 73, 76, 77, 84, 86, 96, 99, 100
Senior professional title	1, 18, 34, 40, 42, 59, 62, 102

**Table 2:** Metrics and conceptual definitions of the social network analysis.

Metric	Conceptual Definition
Density	Proportion of possible relationships in the network. Density values vary between 0 and 1, with 1 being when all possible relationships are present [24].
Centralization	The overall integration or consistency of the graph [25].
Geodesic distance	Measures the extent of connection in the network and is the shortest pathway between two people [26]. The shortest distance between two nodes is measured to obtain a geodesic distance matrix.
Centrality	Includes the three measures Freeman’s degree, closeness, and Freeman’s betweenness. Centrality measures the most connected people by measuring those who interact with the most people [27].
Core–periphery model	Examines the location characteristics of doctors in information dissemination networks [28].
Structural holes	When a social entity is in a position where two other social entities are directly connected, it is in the structural hole position and plays a key connection role. At the same time, in the process of information transmission, the social entity in the structural hole position can access two kinds-of information and can control the group by transmitting and describing this different information. Structural holes include three metrics: effective size, efficiency, and constraint [29].

**Table 3:** Interaction frequency in the e-health community.

	Number of Views	Number of Votes	Number of Favorites
**Average**	80,362	9677	7226
**Median**	55,073	2864	2154
**Moderate**	No	No	No
**Standard**	86,956	21,331	14,212
**Kurtosis**	8	53	23
**Skewness**	2	6	4
**Maximum**	541,639	192,431	104,230
**Minimum**	1359	9	6

**Table 4:** Maximum and minimum of doctor interaction frequency.

	Number of Views	Number of Votes	Number of Favorites
**Maximum**	individual 40	individual 1	individual 62
**Minimum**	individual 13	individual 9/ individual 99	individual 88

**Table 5:** Centralization in the e-health community.

Degree Centralization		Betweenness Centralization	Closeness Centralization	
In-Degree	Out-Degree		In-Degree	Out-Degree
21.95%	32.95%	3.96%	17.17%	27.19%

**Table 6:** Centrality of the doctor information interaction network (partial data)**.**

Node	Freeman’s Degree		Freeman’s Betweenness	Closeness	
	In-Degree	Out-Degree		In-Degree	Out-Degree
1	45	56	476.961	64.331	69.178
2	15	39	58.631	52.880	61.585
3	7	45	132.778	46.119	64.331
8	24	30	97.495	56.742	58.382
10	20	35	107.285	54.891	60.479
12	18	31	88.459	54.595	58.721
31	29	30	163.102	58.382	58.382
34	20	28	100.281	55.495	58.046
35	32	31	150.781	59.412	58.721
36	31	23	94.101	59.064	56.111
39	26	38	158.380	56.742	61.585
40	24	44	167.231	56.425	63.522
42	25	51	196.013	56.111	66.887
44	38	37	168.422	61.585	60.843
47	28	37	114.957	58.046	60.843
53	34	31	118.798	60.119	58.721
56	36	17	59.764	60.843	54.011
58	24	22	48.690	56.425	55.495
59	28	19	56.095	58.046	54.595
60	29	31	112.351	58.046	58.721
61	28	17	55.600	58.046	53.723
62	25	23	69.873	57.062	56.111
66	27	21	52.385	57.062	54.891
68	34	30	101.925	60.119	58.382
69	32	25	101.009	59.412	56.742
73	25	32	136.355	57.062	58.046
84	20	35	95.423	55.495	60.119
86	17	40	118.494	54.595	61.963
87	25	20	52.264	57.062	53.439
92	29	37	188.107	58.382	60.843
96	30	34	146.920	58.382	59.763
100	28	31	136.590	58.046	58.721
102	41	24	158.262	62.733	56.425
